# Radiology report: what is the opinion of the referring physician?

**DOI:** 10.1590/0100-3984.2017.0115

**Published:** 2018

**Authors:** Fernando de Castro Guimarães Rios Ignácio, Luis Ronan Marquez Ferreira de Souza, Giuseppe D’Ippolito, Mayara Martins Garcia

**Affiliations:** 1 Hospital do Coração (HCor), São Paulo, SP, Brazil.; 2 Department of Radiology and Diagnostic Imaging, Universidade Federal do Triângulo Mineiro (UFTM), Uberaba, MG, Brazil.; 3 Department of Diagnostic Imaging, Escola Paulista de Medicina da Universidade Federal de São Paulo (EPM-Unifesp), São Paulo, SP, Brazil.; 4 Instituto de Ciências da Saúde, Universidade Federal do Triângulo Mineiro (UFTM), Uberaba, MG, Brazil.

**Keywords:** Radiology, Radiology information systems, Diagnostic imaging, Radiologia, Sistemas de informação em radiologia, Diagnóstico por imagem

## Abstract

**Objective:**

To evaluate the opinion and perception of referring physicians regarding the radiology report, in order to develop tools that promote an improvement in its quality.

**Materials and Methods:**

We prepared a questionnaire containing ten multiple choice questions about the radiology report, administering it to 70 physicians (35 specialists and 35 residents working in specialties other than radiology).

**Results:**

Referring physicians (specialists and residents) showed a preference for structured reports, with a description explained in universal medical language and a complete conclusion listing the diagnostic possibilities with the degree of certainty. The examination technique should be described, and the final report is best presented when it contains the final chart, together with images, as hard copies and in digital format. The respondents also reported having confidence in the opinion of the radiologist and expressed the need for a direct channel of communication with the same.

**Conclusion:**

Referring physicians seek detailed reports (including a description of the examination technique), preferably structured, with objective language and relevant conclusions (the position of the radiologist on the case is important). It is necessary to discuss the differential diagnoses and provide a form of contact between the parties. Although referring physicians consider the radiologist opinion relevant, they also want to analyze the images on their own.

## Abstract (pt)

**Objetivo:**

Avaliar a opinião e a percepção dos médicos solicitantes sobre o laudo radiológico e desenvolver ferramentas que promovam uma melhora de sua qualidade.

**Materiais e Métodos:**

Foi elaborado um questionário contendo dez questões de múltipla escolha acerca do laudo radiológico, o qual foi submetido a 35 médicos especialistas e 35 médicos residentes de outras especialidades.

**Resultados:**

Médicos referentes (especialistas e residentes) mostraram preferência por laudos estruturados, com uma descrição explicada em linguagem médica universal e conclusão completa contendo as possibilidades diagnósticas indicadas com probabilidade de certeza. A técnica do exame deve ser descrita e o relatório final é mais bem apresentado quando contém o laudo final associado a imagens impressas e em formato digital. Também foram relatadas a confiança na opinião do radiologista e a necessidade de um canal direto de comunicação com ele.

**Conclusão:**

Os médicos solicitantes buscam relatórios detalhados (inclusive com a descrição da técnica do exame), preferencialmente estruturados, com linguagem objetiva e conclusões pertinentes (o posicionamento do radiologista sobre o caso é importante). É necessário discutir os diagnósticos diferenciais e fornecer uma forma de contato entre as partes. Apesar de considerarem relevante a opinião do radiologista, eles também querem analisar as imagens por conta própria.

## INTRODUCTION

The written radiology report is the most important and oftentimes only means of communication between referring physicians and radiologists. It is included in the medical records of patients and plays a fundamental part in the way their clinical care is conducted. The report also incorporates the personal perceptions and abilities of the radiologist. As diagnostic methods become more complex, radiology reports take on an even greater role.

There have been a series of studies evaluating the characteristics of and preferences regarding radiology reports. One recent study demonstrated a certain preference of radiologists for more detailed reports written in free text (i.e., unstructured) and that also include a description of the examination technique employed by the radiologist^(1)^. Another study showed the preference of various imaging centers for reports to be structured in ways that facilitate access to the information, invoicing, teaching, research, and other aspects^(2)^. These and other characteristics form the basis of a radiology report and generate differences of opinion regarding which is the best model to be adopted. However, the opinion of the referring physicians is also crucial to the process of improving radiology reports, given that they will be the final recipients of the reports.

The objective of this study was to evaluate what referring physicians expect from a radiology report and, upon careful evaluation of the results, propose practices that meet their expectations. We highlight the efforts made by The Brazilian College of Radiology and Diagnostic Imaging in this regard, as clearly demonstrated by the creation of a working group on radiology reports^(3)^. The members of the group began their work by examining how radiologists approach the task of preparing their reports. They also attempted to determine in what form the referring physicians preferred to receive those reports. They were then able to devise a series of recommended minimum requirements for the reports^(3)^.

## MATERIALS AND METHODS

This was a prospective, cross-sectional, descriptive study, approved by the local research ethics committee. A questionnaire (Chart 1) composed of ten multiple choice questions was created and sent out (digitally and in print form) to our target audience in each specialized department of the university (internal medicine, surgery, gynecology/obstetrics, pediatrics, and orthopedics). The study sample comprised specialists and resident physicians at a hospital operated by a public university in the southeastern region of Brazil. The study was carried out from October 2015 to March 2016.

**Chart 1:** Questionnaire

**Radiology report**
Dear colleague,
I kindly request that you fill out this questionnaire, which is designed to further scientific research regarding radiology reports in the university sphere. We would like to highlight the fact that this test protects your privacy, and therefore no names are necessary.
Do you agree to participate in our study? YES ( ) NO ( )
Thank you.
1) What is your area of medical specialization?
a) Internal medicine
b) Surgery
c) Orthopedics
d) Other
2) How are you currently situated?
a) Resident
b) Specialist
3) How do you analyze the radiology report for a computed tomography or magnetic resonance imaging examination?
a) I read only the conclusion
b) I read the conclusion and skim the report for relevant findings
c) I read the entire report
d) I don't read the report; I only analyze the images
4) How much do you trust the conclusion in a radiology report?
a) Total trust in the opinion of the radiologist (100%)
b) Partially trust, as an important source of support for secondary decisions (75%)
c) Little (25%), because it is not highly relevant; it simply contains useful information
d) None (0%); I do not use the opinion of the radiologist
5) What is the best way for a radiology report to be presented?
a) Only the final report of the radiologist
b) Final report and printed images
c) Final report and CD
d) The final report with printed images and a CD
6) What is your opinion regarding the use of terminology unique to radiologists?
a) It makes it more difficult to understand the report (it should be simplified)
b) I am able to understand and interpret it with no difficulty
c) It can be used to describe findings, although it should always be accompanied by a conclusion clarifying the meaning of the terminology
d) I have no opinion about it
7) Do you prefer structured or free-text reports?
a) I prefer a structured report (standard format)
b) I prefer a report written in prose (free text)
c) I have no opinion about it
8) What is your opinion about describing the examination technique used in a report (contrast agent and dosage, specifications regarding the equipment, etc.)?
a) I think it's important
b) Better to leave it out (it makes the report unnecessarily long)
c) Indifferent
9) What is your opinion regarding the inclusion of multiple differential diagnoses in a radiology report?
a) It is helpful for making us think of all possible hypotheses
b) Makes things harder, generating confusion. Better to include only the principal hypothesis
c) It helps if the certainty is expressed as a percentage alongside every possible diagnosis
d) I prefer that radiologists do not express their opinion regarding the diagnosis
10) What should a radiologist do when an incidental (unexpected) finding is observed?
a) Converse with the referring physician
b) Describe only in the report
c) Inform the patient
d) Not report anything observed outside the scope of the initial request

The questionnaire was structured in such a way that it could be easily read and completed in only a few minutes. Questions were mainly related to computed tomography and magnetic resonance imaging. Respondent anonymity was guaranteed, and it was therefore not possible to establish the individual characteristics of the participants. From among the completed questionnaires, we selected the first 35 received from specialist physicians (over five years of experience) and an equal number received from resident physicians working in the corresponding specialties (i.e., matched to the specialists). Therefore, the final sample comprised 70 questionnaires completed by physicians.

The statistical analysis of the data collected involved descriptive analysis, through the calculation of absolute and relative frequencies. We also constructed bar charts.

## RESULTS

**Figure 1:**
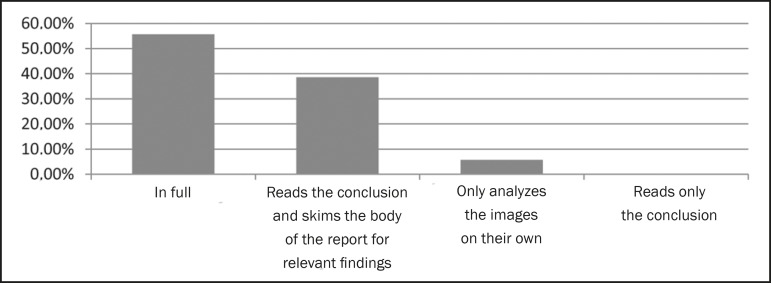
How the report is read and analyzed.

**Figure 2:**
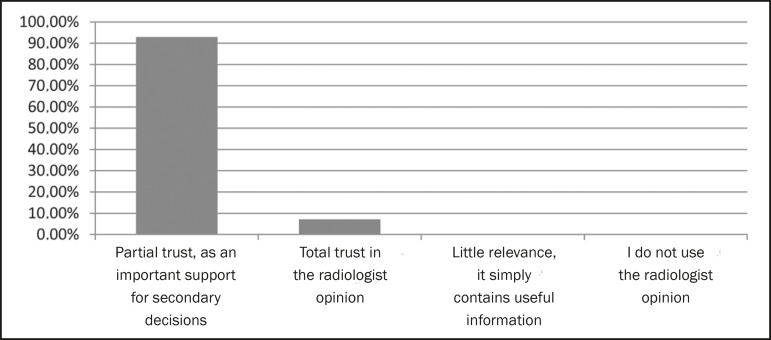
Level of trust in the radiologist opinion.

**Figure 3:**
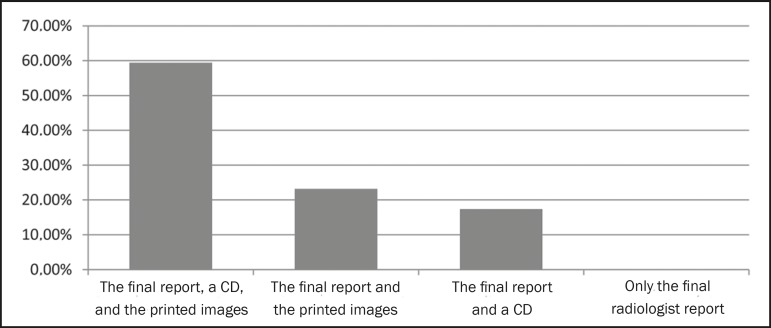
Best form of presentation for a report.

**Figure 4:**
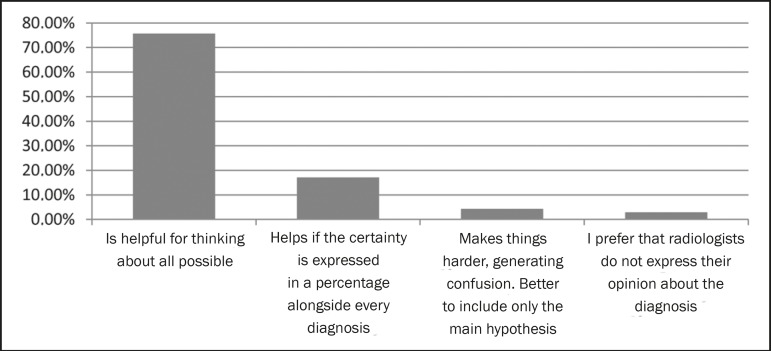
Opinion regarding the inclusion of various differential diagnoses in the conclusion of a report.

**Figure 5:**
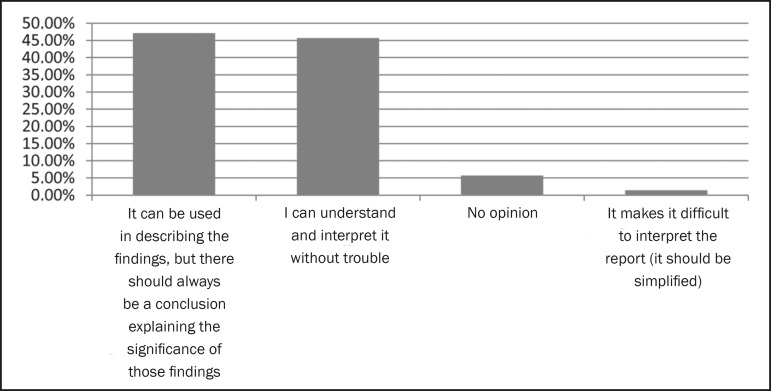
Opinion regarding the use of terminology unique to radiology.

**Figure 6:**
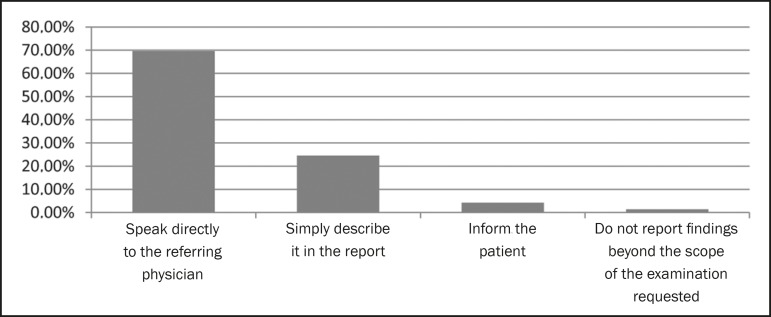
Radiologist practice upon discovering an incidental finding.

Among the respondents, clinicians were the most well represented, accounting for 45.7%. On the basis of the responses, we found that 55.7% of the referring physicians read radiology reports in full (Figure 1), 92.9% trust the opinion of the radiologist only partially (Figure 2), 67.1% prefer that the report be structured, 82.9% prefer that describing the examination technique employed be described, and 59.4% prefer that the images be made available not only as hard copies but also in a digital format (Figure 3). In addition, 75.7% of the respondents stated a preference for conclusions that list different diagnostic possibilities (Figure 4), 47.1% prefer that the technical terminology used in the description be clear (Figure 5), and 69.7% believe that direct communication between the radiologist and the referring physician, either by telephone or in person, is the best practice when there is an incidental finding during an examination (Figure 6).

## DISCUSSION

Radiology reports are the principal means of communication between clinicians and radiologists. A referring physician oftentimes knows a radiologist only from the reports, and radiologists frequently do not know who is on the receiving end of their reports (especially at large health care centers), how they are being evaluated, or what is expected by the referring physicians. Although there have been many studies on radiology reports and their structure, few have researched what referring physicians in Brazil expect regarding such reports.

A careful analysis of our results allows us to conclude that, despite the fact only approximately half of the respondents reported reading radiology reports in full, most of the physicians who request radiology reports at our institution do trust those reports, at least partially, as well as demonstrating a preference for structured reports that describe the examination technique employed and contain objective conclusions. We also observed a clear preference for oral communication between the radiologist and the referring physician.

The European Society of Radiology recently published a reference handbook that highlights the fundamental importance of the conclusion in a radiology report^(4)^, because there is evidence that a large number of physicians do not read the report in full and, in some cases, that will be the only section of the report that will be read. In our study, most of the clinicians (55.7%) indicated that they read the report in full. Another significant proportion of the respondents (38.6%) stated that they read only the conclusion and skim over the report for additional information regarding the findings. Another recent study showed that nearly half of clinicians (46%) read the conclusion and analyze the report further only if complementary information is needed^(5)^. The authors found that only 39% of the participants read the report in full. Another study involving 200 physicians obtained similar results^(6)^. The results of our study, like those of the two abovementioned studies^(5,6)^, show how important it is to construct an appropriate conclusion in a radiology report, given that a significant amount of physicians do not read the report in full.

The aim of the present study was to determine what level of trust referring physicians had regarding radiology reports in a university environment. The vast majority of the respondents (92.7%) indicated that they trust the reports only partially, stating that the reports were used to make secondary decisions, only 7.1% stating that they have complete confidence in the opinion of the radiologist. None of the respondents reported a lack of confidence in or disregard for the radiologist opinion. Our findings corroborate those of recent studies, one of which involved a sample of nearly 4000 physicians, by demonstrating that 87% of referring physicians recognize the fundamental importance of radiology reports, in order to ensure excellence in medical practice^(5,6)^.

According to the American College of Radiology, radiology reports should contain images and multimedia^(2)^. Our study investigated what is the best way in which to present the results of an examination, in the opinion of referring physicians. Most of the referring physicians surveyed (59.4%) stated a preference for the final report to be accompanied by printed photos and a CD with digital images. Another study also demonstrated that physicians working in university hospitals, a sample comparable to ours, prefer to receive the report accompanied by a CD or DVD, whereas those working in public hospitals prefer printed photos^(5)^. Making images available online is a new form of presentation that has also been gaining ground, because it reduces the costs of material and manpower, as well as making life easier for the patient^(9)^. However, that form of presentation was not covered in our questionnaire, given that this option for sharing data is still not available at our facility.

A recently published study^(10)^ identified clarity as one of the facets referring physicians consider to be most important in radiology reports. In another study^(6)^, which included only X-ray reports, 39% of referring physicians stated that the report was confusing and 51% stated that the initial reason for the examination to be requested was not covered at all. Clarity is of essence in radiology reports, because it guarantees that the information being transmitted is precise and completely intelligible, thus directly benefitting the patient. Therefore, we choose to evaluate the technical language used by radiologists. A considerable proportion (47.1%) of the referring physicians surveyed in the present study believe that although technical terminology can be used in describing the findings, the report should include a conclusion from the radiologist explaining the significance of those findings. However, approximately half of the studied population reported having difficulty in understanding the language employed in radiology reports. Two studies, both conducted in 2011, presented diverging results on the subject. In one of those studies, 77.5% of referring physicians reported having no trouble understanding what the radiologist was attempting to communicate^(7)^, whereas the results of the other study suggest the need to create a universal medical language to be used in radiological reporting^(5)^. That divergence is reflected in our results, evidenced by the proximity of the proportions for two most common responses. Therefore, it is clear that a significant proportion of referring physicians find it challenging to understand the technical terminology used by radiologists, which makes is necessary to, at least in the conclusion, employ medical terminology that is a more universally understood.

The American College of Radiology and European Society of Radiology both recommend that radiology reports be structured (divided into ordered sections) and employ standard terminology, in order to improve the way in which the results of a radiological procedure are communicated, as well as that the reports make information easier to recover and reuse^(2,4)^. In the present study, we also evaluated the opinion of referring physicians regarding the way in which reports are structured and found that the vast majority of those physicians (67.1%) prefer structured reports to free-text reports, which were preferred by only 17.1% of the respondents. Other studies have reported similar findings^(1,7,11)^.

Various studies have demonstrated that the format of the report has no significant impact on reading time and comprehension^(12-14)^. The literature also demonstrates that, in comparison with free-text reports, structured reports can reduce accuracy and thoroughness^(15)^. One recent study surveyed radiologists from different countries who had gathered for a European congress on radiology^(16)^. Most of those radiologists stated that the reports issued at the facilities where they work are already structured to a certain extent, and that the adoption of fully structured reports is impaired due to the complexity of the task of preparing such reports and the impact that they would have on the productivity of the facility. That same group of radiologists demonstrated a clear preference for what could be considered a semistructured report^(16)^.

Describing the radiological technique used in an examination is considered a fundamental element of the preparation of a high-quality report. The results obtained from our survey revealed that the vast majority (82.9%) of referring physicians understand that it is important to describe the examination techniques (type/dose of contrast agent administered, type of equipment employed, etc.) in the report. Our results are in line with those of other studies, which have also shown that there is a preference for the technical aspects to be described in a report^(1)^.

Vague reports with ambiguous wording, in which the radiologist does not take responsibility for a diagnosis, are questioned by many referring physicians^(16)^. Within that context, our study investigated whether referring physicians preferred a direct conclusion leading to a single diagnosis or multiple plausible diagnoses for a specific case. The majority (75.7%) preferred that a range of differential diagnoses be described in the conclusion. Another group (17.1%) also considered the reporting of diverse diagnoses to be a positive thing, provided that the percentage certainty for each diagnostic possibility be displayed as well. That is still a rare practice in Brazil^(10)^. Other studies corroborate the participant preference for the inclusion of various differential diagnoses while also emphasizing the importance of clearly stating the degree of certainty for each hypothesis^(16,17)^, a percentage being the best way to express that^(10,18)^. The suggested maximum number of diagnostic possibilities to be included in a report is three; if there are more than three, the examination should be repeated^(17)^.

There are various situations in which it will be necessary for the radiologist and referring physician to confer, such as when there is a relevant incidental finding. In our study, we attempted to determine what would be the best way to conduct that consultation. The majority (69.6%) of the referring physicians felt that the radiologist should converse with the referring physician directly, either in person or over the telephone. Other studies have also demonstrated that referring physicians prefer to have a direct line of communication with the radiologist, stating that it is a top priority^(5,16)^.

Our study has certain limitations. The sample was relatively small, and all of the participants worked at the same health care facility in the same city. In addition, we did not perform an epidemiological analysis (of the age, gender, training, etc.) of the professionals who participated in the study. An analysis of those aspects could reveal disparities between or among regions, professionals, (in terms of the level of experience), and genders.

After analyzing the data collected in our study, we concluded that the referring physician gives considerable weight to the opinion of the radiologist, underscoring the importance of radiologists in ensuring excellence in medical practice. There is a preference for reports that are structured, are clear, and offer a simplified explanation of the radiologist terminology, as well as containing a description of the examination technique employed. The conclusion of the report should receive special attention, being that it is oftentimes the first (if not the only) section to be read. Various diagnostic possibilities should be laid out by the radiologist and, when possible, should be accompanied by the corresponding degree of certainty. The report presented to the referring physician should contain the final report, together with printed and digital images. Finally, whenever it is necessary for radiologists and the referring physicians to communicate, there should be direct contact between the two.
